# *N*- and *O*-glycans in Unfertilized Chum Salmon (*Oncorhynchus keta*) Eggs Using Glycomic Techniques

**DOI:** 10.3390/ijms27104646

**Published:** 2026-05-21

**Authors:** Masaki Kurogochi, Kai Suzuki, Di Wu, Hisatoshi Hanamatsu, Ken Kitajima, Chihiro Sato, Jun-ichi Furukawa

**Affiliations:** 1 Institute for Glyco-core Research (iGCORE), Nagoya University, Tokai National Higher Education and Research System, Furo-cho, Chikusa-ku, Nagoya 464-8601, Japan; suzuki.kai.h4@s.mail.nagoya-u.ac.jp (K.S.); wu.di.u5@f.mail.nagoya-u.ac.jp (D.W.); kitajima.ken.k8@f.mail.nagoya-u.ac.jp (K.K.); sato.chihiro.r9@f.mail.nagoya-u.ac.jp (C.S.); 2 Graduate School of Bioagricultural Sciences, Nagoya University, Tokai National Higher Education and Research System, Furo-cho, Chikusa-ku, Nagoya 464-8601, Japan; 3 Department of Orthopaedic Surgery, Faculty of Medicine and Graduate School of Medicine, Hokkaido University, Kita-15, Nishi-7, Kita-ku, Sapporo 060-8638, Japan

**Keywords:** *N*-glycan, *O*-glycan, polysialic acid, sulfated glycan, unfertilized eggs, MALDI-TOF MS

## Abstract

Genomic analysis of various fish has advanced in recent years; however, predicting glycan information from the genomic data alone remains challenging. Glycomic techniques have therefore attracted considerable attention. In this study, we analyzed *N*- and *O*-glycans in unfertilized *Oncorhynchus keta* eggs using glycomic techniques, such as a glycoblotting procedure, a sialic acid linkage-specific alkylamidation, and an evaporative β-elimination with pyrazolone. *N*-Glycomic analysis revealed that biantennary *N*-glycans were predominant, and that sialylation occurred via an α2,3 linkage. In addition, numerous sulfated *N*-glycans were observed, some of which had not been reported previously. Tandem mass spectrometry analyses indicated that most of the sulfate groups were attached to GlcNAc linked to mannose within the core structure. The sulfation sites of the unknown sulfated glycans were the same; however, a GlcNAc residue was lost from the core structure. In *O*-glycomics, oligo-sialylated *O*-glycans, which contain between one to seven sialic acid residues, were observed in the unfertilized eggs. Most of the sialic acids in the *O*-glycans were Neu5Gc, and there was no α2,3 linkage.

## 1. Introduction

Glycans bind to lipids or proteins and are expressed on the surfaces of normal and mutant cells [1]. They regulate cell–cell interactions and mediate signal transduction in various biological processes [2]. Recent advances in mass spectrometry (MS) and glycoscience have enabled glycome research on human health and disease, elucidating the functions and diversity of glycans. Furthermore, these technologies have expanded the characterization of various glycans in non-human organisms. Fish are recognized as valuable marine resources, and research in aquaculture and fish glycomics is advancing our understanding of reproductive and disease-related processes. Glycomic research is progressing in various species of fish, including model organisms such as zebrafish (*Danio rerio*) [3,4], medaka (*Oryzias latipes*) [5,6], Atlantic salmon (*Salmo salar*) [7,8,9], rainbow trout (*Oncorhynchus mykiss*) [10,11,12], flounder (*Paralichthys olivaceus*) [13], freshwater trout (*Plecoglossus altivelis*) [14], and Atlantic cod (*Gadus morhua*) [15]. Recently, numerous integrated glycomic studies analyzing two or more glycan classes from biological samples have been reported [16]. This approach is expected to advance our understanding of the role of glycoconjugates in various biological processes. Yamakawa et al. [4] reported the precise glycomic profiles of eight individual zebrafish organs, including *N*-glycans, *O*-glycans, and glycosphingolipids. These glycans were permethylated and analyzed using Matrix-Assisted Laser Desorption/Ionization Time-of-Flight mass spectrometry (MALDI-TOF MS). It is known that *O*-glycans containing α2,8-linked oligosialic acid are present in the unfertilized eggs of salmon and rainbow trout [17,18]; however, analysis of *N*-glycans remains limited [19], and no detailed studies have yet been reported.

We developed the sialic acid linkage-specific alkylamidation (SALSA) method, which utilizes differential derivatizations with methylamine or isopropylamine to discriminate between α2,3/2,8- and α2,6-linked sialic acids. The amidations of carboxyl groups in sialic acid suppress their decomposition during the ionization process in MALDI-TOF MS analysis, resulting in high-sensitivity detection comparable to that of neutral glycans. However, the SALSA method has not been used to measure glycans that include three or more sialic acid residues linked via α2,8 linkages.

**Figure 1:**
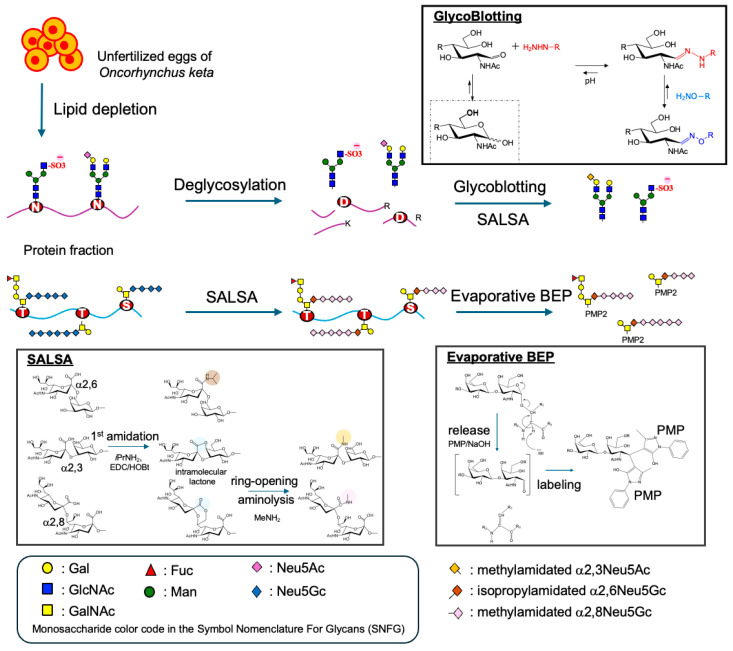
Procedure for the glycomics of *N*- and *O*-glycans in the unfertilized eggs of *Oncorhynchus keta*.

In this study, we aimed to clarify the glycan structures between both *N*- and *O*-glycans of glycoproteins, which are the predominant post-translational modifications, in the unfertilized chum salmon (*Oncorhynchus keta*) eggs. The *N*- and *O*-glycans were prepared from the unfertilized eggs using the glycoblotting technique [20] and evaporative β-elimination with the pyrazolone (BEP) method [21], both of which involve the aminolysis-SALSA procedure [22] (Figure 1). In MALDI-TOF MS analysis, most *N*-glycans were bi-antennary complex structures. Notably, *N*-glycans were found to contain negatively charged sulfate groups by MALDI-TOF MS and tandem mass spectrometry (MS/MS) analyses. The SALSA method revealed that most *O*-glycans consisted of α2,6-linked sialic acid bound to a GalNAc residue at the reducing end and further elongated into oligosialic acid with α2,8-linked sialic acids. In contrast, small amounts of α2,3-linked sialic acids were present in *N*-glycans.

## 2. Results

### 2.1. Analysis of N-glycans in Unfertilized O. keta Eggs

**Figure 2:**
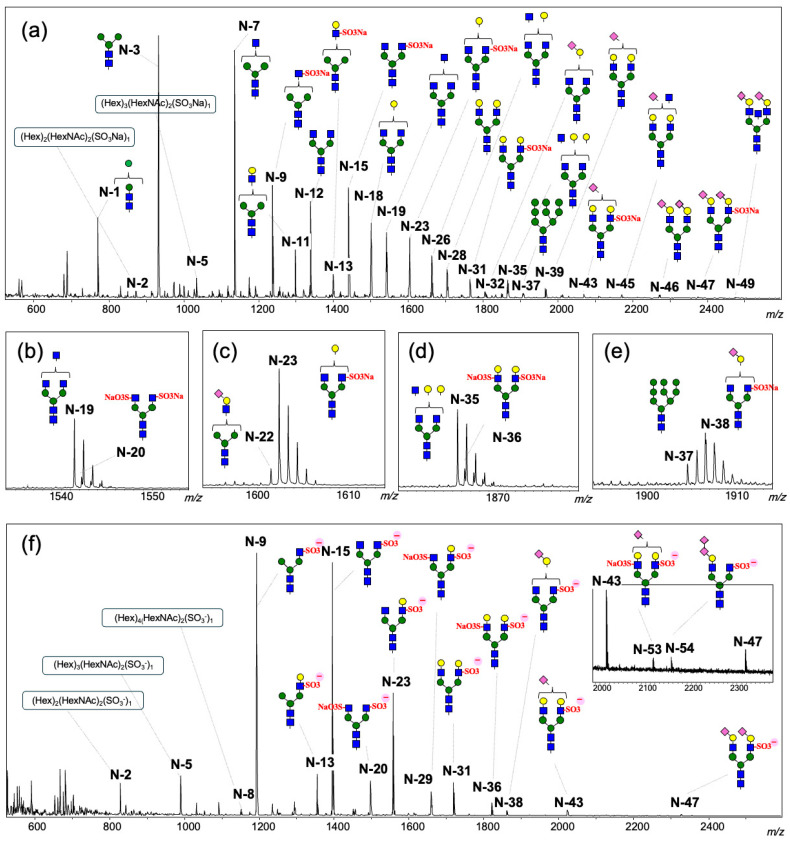
MALDI-TOF MS spectra showing *N*-glycans in unfertilized *Oncorhynchus keta* eggs (**a**) full view and enlarged view of (**b**) N-19 and N-20, (**c**) N-22 and N-23, (**d**) N-35 and N-36, and (**e**) N-37 and N-38 in positive ion mode. (**f**) full view in negative mode.) Signal numbers correspond to those described in Table 1 and Table S1. Putative glycan is illustrated with the schematic diagram of the SNFG (Shown in Figure 1).

**Figure 3:**
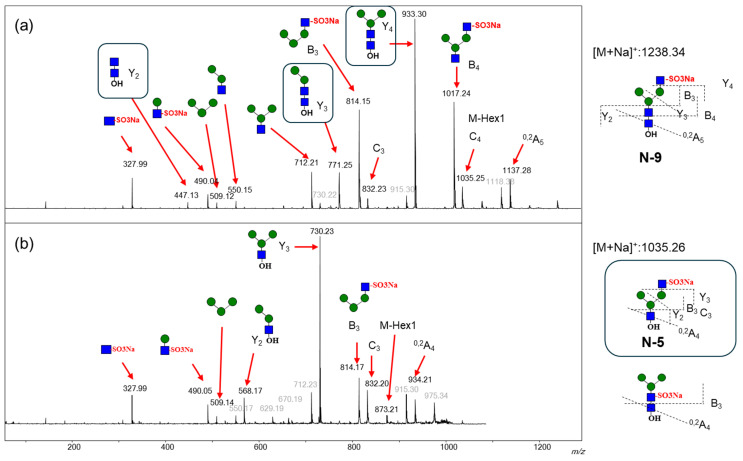
MALDI-TOF/TOF MS spectra of negatively charged monosulfated *N*-glycans at (**a**) *m*/*z* 1238.24 (N-9), and (**b**) 1035.26 (N-5). Putative glycan is illustrated with the schematic diagram of the SNFG (Shown in Figure 1).

Previous sugar composition analyses have reported the existence of complex and hybrid *N*-glycans in egg proteins from three salmonid genera (*Salmo*, *Salvelinus*, and *Oncorhynchus*); however, their detailed structures have not been elucidated [19]. A detailed structural analysis of *N*-glycans in unfertilized *O. keta* eggs is still lacking. To address this, we analyzed *N*-glycans in unfertilized *O. keta* eggs using the glycoblotting [20] and aminolysis-SALSA methods [22]. A total of 49 *N*-glycans were detected as sodium adduct ions, and the neutral complex-type *N*-glycans were major components by MALDI-TOF MS in positive ion mode (Figure 2a, Table 1). The SALSA method was used to detect sialylated *N*-glycans, revealing that the predominant sialic acid linkage was α2,3-linked Neu5Ac. Notably, *N*-glycans containing sulfate groups were detected as positively charged ions bearing sodium sulfate, including 12 types of monosulfated and 3 types of disulfated *N*-glycans. The results were generally reproducible across three independent measurements (Table 1). While sulfate (SO_3_H: 79.9568) and phosphate (PO_3_H_2_: 79.9663) groups have almost the same molecular weight, they can be distinguished in positive ion mode by their distinct salt adducts: SO_3_Na (101.9388), PO_3_Na_2_ (123.9302), and PO_3_NaH (101.9483) [23]. The N-9 signal was initially attributed to either the mono-sulfated or mono-phosphated sodium adduct of N-7; however, no di-sodium adduct was detected. Consequently, the absence of the di-sodium adducts confirmed that the modification was derived from a sulfate group rather than a phosphate group. Additionally, disulfated glycans can be detected using MALDI-TOF MS. They were detected as sodium-adducted glycan ions with two uncharged SO_3_Na ions (Table 1). We evaluated the reproducibility of detecting sulfated *N*-glycans in positive ion mode using purified, 2-AB-labeled glycans of known concentration. As shown in Figure S1, high reproducibility was maintained across various mixing ratios of neutral and sulfated glycans, with coefficients of variation (CVs) consistently below 2%. Although the signal intensity of sulfated glycans was approximately 20–28% of that of neutral glycans and ISD (in-source decay) occurred due to the loss of sulfate groups, the sensitivity remained sufficient for the quantitative analysis of sulfated glycans. Interestingly, the signals of sulfated (Hex)_2_(HexNAc)_2_, (Hex)_3_(HexNAc)_2_, and (Hex)_4_(HexNAc)_2_ were observed. While sulfate groups in complex- and hybrid-type *N*-glycans have been reported to be attached to the non-reducing terminal Gal and GalNAc residue of *N*-acetyllactosamine and *N*, *N*′-diacetyllactosediamine, GlcNAc residues attached to mannose in the core structure are sulfated. However, no reports have been published regarding sulfate groups attached to paucimannose-type *N*-glycans [24,25,26]. Therefore, we performed MS/MS analyses of these sulfated glycans to identify their structures. MS/MS spectra of sulfated N-glycans N-9 [(GlcNAc)_1_ + (Man)_3_(GlcNAc)_2_ + (SO_3_Na)_1_] and N-5 [ (Hex)_3_(HexNAc)_2_ + (SO_3_Na)_1_] are shown in Figure 3a. The ion thought to be sulfated complex-type *N*-glycan [N-9, *m*/*z* 1238.34 (Hex)_3_(HexNAc)_3_(SO_3_Na)_1_ + Na^+^] produced an ion at *m*/*z* 327.99; (HexNAc)_1_(SO_3_Na)_1_ + Na^+^, indicating that the sulfate group is attached to the GlcNAc residue rather than the Man residue. Additionally, the Y2 (*m*/*z* 447.13), Y3 (*m*/*z* 771.25), and Y4 (*m*/*z* 933.30) fragment ions confirmed the structure of a complex type with a non-sulfated core structure, resulting in a sulfated HexNAc at the non-reducing end. As shown in Figure 3b, the ion thought to be a sulfated glycan [N-5, *m*/*z* 1035.26 (Hex)_3_(HexNAc)_2_(SO_3_Na)_1_ + Na^+^] produced the same ion regarding the (HexNAc)_1_(SO_3_Na)_1_ + Na^+^ (*m*/*z* 327.99) fragment. However, the Y2 (*m*/*z* 549.10) and Y3 (*m*/*z* 873.21) fragment ions corresponding to (GlcNAc)_2_ and (Man)_2_(GlcNAc)_2_ of N-9 were not detectable, and the fragment ions at *m*/*z* 568.17 and 730.23 were clearly present.

Next, *N*-glycans of unfertilized eggs were treated by endo-CC enzymes to confirm the detailed structures of N-2, N-5, and N-8. As shown in Figure S2, the signal of N-15 was significantly reduced after the treatment of endo-CC, generating (Hex)_3_(HexNAc)_3_(SO_3_Na)_1_ (N-9 isomer), which has the same glycan composition as N-9.

The signal of N-5, which was digested from N-9, increased. The fragment pattern of N-5 remained unchanged after the treatment of endo-CC (Figure S3a,b). However, the fragmentation pattern of the N-9 isomer differed from that of endogenous N-9 (Figure S3c,d). The distinctive fragment ions at *m*/*z* 568.11 and 837.14, according to Y2 − HexNAc and Y2 + SO_3_Na, were observed in the N-9 isomers. These results indicate that N-5 lacked GlcNAc at the reducing end, which was probably cleaved by endogenous endoglycosidase. Additionally, the detection of sialylated *N*-glycans indicated that the primary sialic acid structure in *N*-glycans was α2,3-linked Neu5Ac, as per the SALSA method.

We also measured *N*-glycans derived from the unfertilized eggs in negative ion mode. Only *N*-glycans bearing negatively charged sulfate ions (SO_3_^−^) were detected (Figure 2b, Table S1). Fifteen types of monosulfated *N*-glycans and five types of disulfated glycans were detected, including fifteen sulfated *N*-glycans in positive ion mode. In negative ion mode, sulfated glycans are ionized by negatively charged SO_3_^−^ ions (80 Da) attached to uncharged glycans, whereas in positive ion mode, they are ionized by positively charged sodium ions attached to glycans containing uncharged SO_3_Na (102 Da). In the positive ion mode of MS analysis, the peaks of sulfated glycans (N-20, -23, -36, and -38) overlapped with those of neutral glycans (N-19, -23, -35, and -37), which rendered identification of the glycan composition difficult (Figure 2b–e). However, sulfated glycans are easily identified in the negative ion mode due to ionization with only negatively charged glycans.

### 2.2. Analysis of O-glycans in Unfertilized O. keta Eggs

Polysialoglycoproteins (PSGPs) with *O*-glycans containing oligo/polysialic acid have been discovered in the unfertilized eggs of *Salmo gairdneri* and other salmonid fish [17,18]. The SALSA method enables the differentiation of α2,3/2,8- and α2,6-linked sialic acid based on their different molecular weights. However, the SALSA method has never been used to analyze glycans that include three or more sialic acid residues with an α2,8 linkage.

**Figure 4:**
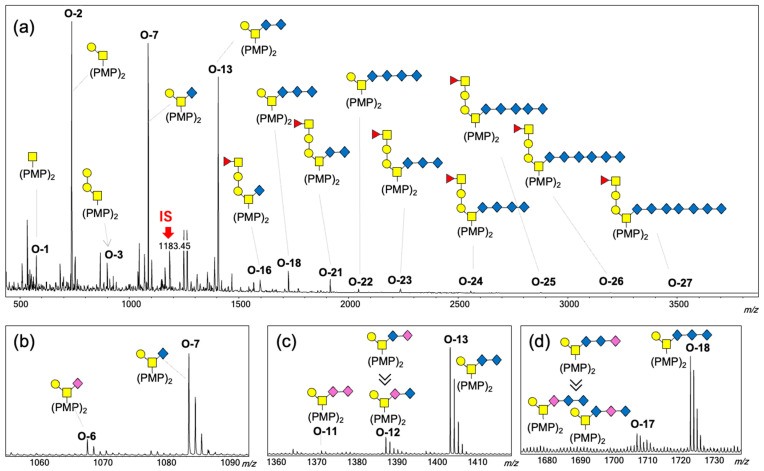
MALDI-TOF spectra of *O*-glycans in unfertilized *Oncorhynchus keta* eggs in positive ion mode ((**a**) full view and enlarged view of (**b**) monosialyl, (**c**) disialyl, and (**d**) tri-sialyl T antigen). Signal numbers correspond to those described in Table 2. The red arrow indicates the internal standard glycan (GN4) peak. Putative glycan is illustrated with the schematic diagram of the SNFG (Shown in Figure 1).

**Figure 5:**
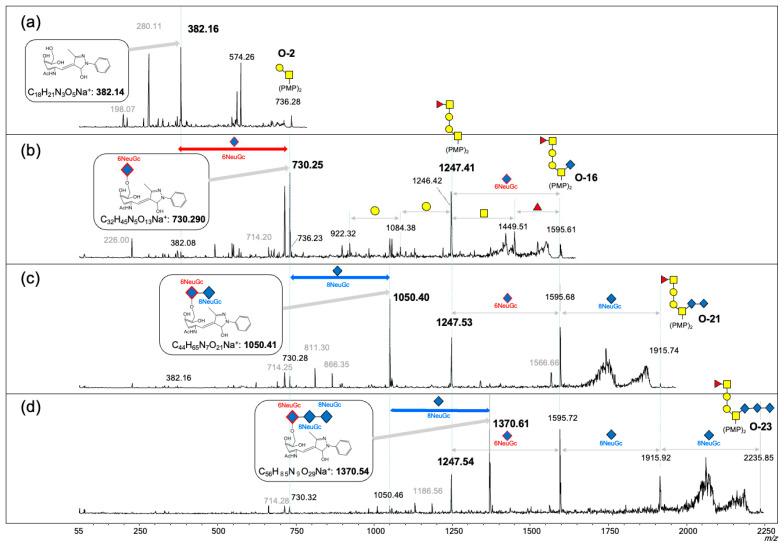
MALDI-TOF/TOF MS spectra of *O*-glycans at (**a**) *m*/*z*: 736.28 (O-2), (**b**) 1595.61 (O-16), (**c**) 1915.74 (O-21), and (**d**) 2235.85(O-23). Putative glycan is illustrated with the schematic diagram of the SNFG (Shown in Figure 1).

Therefore, we analyzed commercially available colominic acid using the SALSA procedure. The colominic acids were directly derivatized via in-solution aminolysis-SALSA [22] and analyzed using MALDI-TOF MS in linear mode. The spectrum of non-derivatized colominic acids showed low sensitivity, with a maximum of 15 sialic acid repeats observed. In contrast, colominic acids were successfully derivatized using SALSA and detected with high sensitivity, reaching up to the 36-mer level (Figure S4). We then investigated the *O*-glycan preparation from unfertilized *O. keta* eggs using the direct-SALSA method and evaporative BEP method [21]. The carboxyl groups of the sialic acid residues on glycans were directly amidated using in-solution aminolysis-SALSA. The *O*-glycans were then cleaved from proteins and labeled with PMP using evaporative β-elimination. Consequently, the bis-PMP labeled *O*-glycans were purified using a C18 SPE column and analyzed by MALDI-TOF MS in positive ion mode. In total, 27 types of *O*-glycans containing oligosialic acid structures were detected as sodium adduct ions (Figure 4, Table 2). A majority of these glycans were Neu5Gc. Oligosialic acids with a maximum of seven subunits (heptamer) can be detected. Reportedly, the oligosialic acids in PSGP extend from an initial Neu5Gc residue attached to the C6 position of GalNAc at the reducing end [17,18]. MS/MS analysis of O-16 (*m*/*z* 1595.61) and its sialic acid-extended *O*-glycans (O-20, O-23, O-24, O-25, O-26, and O-27) revealed the common structure of Fuc-GalNAc-Gal-Gal-GalNAc. MALDI-TOF MS/MS spectra of T-antigen (O-2) and the oligosialic *O*-glycans (O-16, O-21, and O-23) are shown in Figure 5. Similarly, MS/MS measurements of *O*-glycans with the T-antigen (O-2) generated fragments that exhibited branched structures (Figure 5a). MS/MS analysis of *O*-16 showed a distinctive fragment ion at *m*/*z* 730.25, suggesting that Neu5Gc was linked to the C-6 position of GalNAc (Figure 5b). It is suggested that this distinctive fragment ion (730 Da) was formed by the cleavage of a PMP residue (174 Da) and glycan (673 Da) bound to C-1 and C-3 positions of the GalNAc at the reducing end, followed by dehydration (18 Da). Both O-21 and O-23 had an extended Neu5Gc with α2,8 linkage (320 Da) from O-16 and O-21, respectively (Figure 5c,d). Our MS analysis showed that *O*-glycans present at high concentrations contain only trace amounts of Neu5Ac. (Figure 4b–d). The direct-SALSA method and evaporative BEP method have been demonstrated to be effective in determining the structure of oligosialic acid.

## 3. Discussion

Fish glycomics has been conducted in numerous species (*Salmo salar* [7,8,9], *Oncorhynchus mykiss* [10,11,12], *Paralichthys olivaceus* [13], *Plecoglossus altivelis* [14], and *Gadus morhua* [15]) and experimental model organisms such as *Danio rerio* [3,4] and *Oryzias latipes* [5,6]. Although genomic analysis of these fish is progressing, glycans are affected by various environmental factors. Therefore, genomic data alone cannot predict the glycans that serve as biomarkers of reproduction or disease. Consequently, glycomics has attracted significant attention in recent years, as it provides unique insights that differ from genomic analysis. Previous research has examined *O*-glycans in the skin of Atlantic salmon, highlighting how geographical location and body size at various life stages contribute to individual glycan profiles [9]. These studies demonstrated clear regional differences, yet minimal individual variation was observed within the same geographical area. In the present study, our analysis was limited to an unfertilized egg from a single salmon specimen collected in Hokkaido, Japan. Consequently, we are unable to provide data reflecting regional or life-stage-specific variations. However, the glycan structure identified in our analysis is consistent with previously reported structures, and we anticipate that these findings will serve as a valuable reference for future cohort studies.

While *N*-glycans in the eggs of *Oryzias latipes*, *Plecoglossus altivelis*, and *Tribolodon hakonensis* have been previously characterized [27], the characterization of those in salmon eggs has largely been lacking. For instance, although the *N*-glycosylation of phosvitin—a major component of unfertilized eggs from the genera *Salmo*, *Salvelinus*, and *Oncorhynchus*—has been previously reported [19] and sugar composition analysis suggested the presence of complex and hybrid types, their detailed structures remain to be fully elucidated. It is now known that *N*-glycans in eggs of several fish species, excluding salmonids, predominantly consist of complex types with bi-, tri-, and tetra-antennary chains containing α2,3–linked Neu5Ac [3,5,6,13,14,15,24]. In this study, we performed a detailed analysis of both *N*- and *O*-glycans in unfertilized *O. keta* eggs using glycoblotting [20], aminolysis-SALSA [22], and the evaporative BEP methods [21]. *N*- and *O*-glycans—post-translational modifications of glycoproteins—in unfertilized eggs (whole cells) were analyzed using highly sensitive MALDI-TOF and -TOF/TOF MS. In unfertilized *O. keta* eggs, bi-antennary complex-type *N*-glycans were observed, whereas tri- and tetra-antennary structures were not detected. The sialic acid present was Neu5Ac with an α2,3 linkage, consistent with findings in other fish eggs. A substantial number of sulfated *N*-glycans—which have not been previously reported in other fish eggs—were detected. This likely reflects recent advancements in MS technology and preparation methods, which enable highly sensitive and reproducible detection of sulfated glycans. However, because sulfated glycans exhibit lower signal intensities compared to neutral glycans, their quantitative analysis remains a persistent challenge. Sulfated glycans have been observed in the protein components (ovomucoids) of chicken eggs [28]. Generally, the sulfated sites of complex-type *N*-glycans are either GlcNAc residues at the branched site attached to mannose or GalNAc residues at the non-reducing end [24,25,26]. MS/MS analysis showed that the sulfate group was bound to the GlcNAc residue that extended from the core structure of the *N*-glycan. Free *N*-glycans, which were probably cleaved by endogenous endoglycosidase, were found to be present. Additionally, di-sulfated biantennary complex *N*-glycans were detected. Each sulfate group was also bound to each GlcNAc residue extending from the core structure of the *N*-glycan.

*O*-glycan analysis demonstrated that unfertilized *O. keta* eggs contained a characteristic oligosialic acid structure similar to that of *Oncorhynchus mykiss* and other salmonid fish [17,18]. Polysialic acid is highly unstable during MS analysis. While permethylated polysialic acid has been measured using MALDI-TOF MS [29], this study is the first to measure SALSA-derivatized oligosialic acid by MALDI-TOF MS. Our previous work demonstrated that SALSA-derivatized *O*-glycans can be detected with high sensitivity and quantitative accuracy without any degradation, compared to non-derivatized *O*-glycans [21]. These findings suggest that the SALSA method is also highly suitable for analyzing oligo- and polysialic acid structures. MS and MS/MS measurements revealed that most of the sialic acids in the *O*-glycans were Neu5Gc, with small amounts of Neu5Ac-containing *O*-glycans. Monosialyl, disialyl, and trisialyl T-antigens were present in high concentrations in unfertilized eggs, with approximately 10–15% of total sialic acids (Figure 4b–d). By extracting and analyzing *N*- and *O*-glycans from unfertilized eggs, we compared their structural data. Notably, sialic acid composition in *N*-glycans differs from that in *O*-glycans. The sialic acid in *O*-glycans is primarily Neu5Gc, whereas that in *N*-glycans includes Neu5Ac. Neu5Gc is synthesized by cytidine monophosphate (CMP)-Neu5Ac hydroxylase [30]. The hydroxylation of CMP-Neu5Ac is a multi-component reaction catalyzed by cytochrome b5, cytochrome b5 reductase, and CMP-Neu5Ac hydroxylase in the presence of NADH [31,32]. Even in animals expressing Neu5Gc, it was not detected in nervous system cells, indicating a tissue-specific inhibitory mechanism of the CMP-Neu5Ac hydroxylase family [33]. The difference in sialic acid content between *N*- and *O*-glycans in unfertilized *O. keta* eggs indicates a site-specific mechanism governing their biosynthesis within a single cell. Given the potential involvement of various factors, further research addressing these variables is required.

We demonstrated that integrated glycomics allows both *N*- and *O*-glycans to be analyzed simultaneously, thereby showing its potential to investigate the interaction networks across various glycan classes throughout the whole cell. Glycosylation, including sulfation, varies significantly between species and across cell types within a species, indicating that glycan diversity may have contributed to animal evolution. Animal glycomics will increasingly reveal the patterns and mechanisms shaping glycome diversity during animal evolution. We have conducted comprehensive glycomic analysis encompassing glycoproteins, proteoglycans, glycolipids, and free oligosaccharides. We expect that integrated glycomic research focusing on glycan networks, incorporating various cohort studies, will contribute to a deeper understanding of the mechanisms underlying reproduction and disease in fish.

## 4. Materials and Methods

### 4.1. Materials

Tris (2-carboxyethyl) phosphine hydrochloride (TCEP) and 2,5-dihydroxy benzoic acid (DHB) were purchased from Sigma-Aldrich (St. Louis, MO, USA). Phosphate-buffered saline (PBS) was purchased from FUJI FILM Wako Pure Chemical Corporation (Osaka, Japan). 1-Phenyl-3-methyl-5-pyrazolone (PMP) was purchased from Tokyo Chemical Industry (Tokyo, Japan). The SialoCapperID Kit was obtained from Shimadzu Corporation (Kyoto, Japan). BlotGlyco^®^ beads were obtained from Sumitomo Bakelite Co., Ltd. (Tokyo, Japan). Peptide-N-glycosidase F (PNGase F) primer was purchased from N-Zyme Scientifics (Doylestown, PA, USA). Unfertilized *O. keta* eggs were purchased from a supermarket in December.

### 4.2. Sample Preparation

#### 4.2.1. Extraction of Glycoprotein Fractions from Salmon Eggs

Unfertilized *O. keta* eggs (1.65 g) were homogenized with a stirring rod and ultrasonication, mixed with cold acetone (10 mL), and centrifuged (3000× *g*, 5 min) to collect the precipitate. The precipitate was dissolved in 1 mL PBS, followed by the addition of cold acetone (10 mL) and centrifugation (3000× *g*, 5 min). This procedure was repeated three times to obtain a protein fraction.

#### 4.2.2. *N*-glycan Release

The protein fraction from unfertilized eggs (500 µg) was mixed with 10 mM TCEP in 100 mM NH_4_HCO_3_ and reduced at room temperature for 60 min. Subsequently, the mixture was alkylated with 22 mM iodoacetamide at room temperature for 30 min in the dark. The alkylated proteins were digested with trypsin (50 µg) in 50 mM NH_4_HCO_3_ at 37 °C for 3 h, followed by heat inactivation of the enzyme at 90 °C for 15 min. After cooling, the *N*-glycans were released from the tryptic glycopeptides by incubation with PNGase F Prime (334 U) at 37 °C for 6 h.

#### 4.2.3. Purification of *N*-glycan Using Glycoblotting and SALSA Procedure

Glycoblotting of sample mixtures containing released *N*-glycans was performed as previously described [20]. BlotGlyco^®^ beads (500 µL) (10 mg/mL suspension) were aliquoted into the wells of a polytetrafluoroethylene 96-well filter plate. After removing water using a vacuum pump, 20 µL of PNGase F-digested samples were applied to the wells, followed by the addition of 180 µL of 2% acetic acid (AcOH) in acetonitrile (ACN). Subsequently, the plate was incubated at 80 °C for 45 min to capture the *N*-glycans on the beads through a chemically stable and reversible hydrazone bond. The beads were washed with 300 µL of 2 M guanidine-hydrogen chloride (HCl), followed by washing with the same volume of water and 1% triethyl- amine in methanol (MeOH). Each washing step was performed twice. Subsequently, *N*-glycan-linked beads were incubated with 10% acetic anhydride in MeOH (100 µL) for 30 min at room temperature to cap unreacted hydrazide groups by acetylation. Following capping, sialylated *N*-glycans were derivatized using the SALSA procedure on a solid support. The beads were incubated with 100 µL of premixed Reagents A and B from the SialoCapper™-ID Kit (Shimadzu Corporation, Kyoto, Japan) for 1 h at room temperature using a shaking plate mixer [22]. After the reaction, the beads were washed twice with MeOH and Reagent C from the SialoCapper™-ID Kit. The beads were serially washed with 200 µL of MeOH, 100 mM HCl, and water, with each step performed twice. The captured glycans were released with an 8% AcOH solution (150 µL) at 80 °C for 30 min. As an internal standard for relative quantitative analysis, 500 pmol of A2GN1 was added before the glycoblotting procedure. After the reaction, the derivatized *N*-glycans were recovered by filtering the solution and then washing the beads with water (100 µL).

#### 4.2.4. Preparation of In-Solution SALSA-Derivatized *O*-glycan Using the BEP Method

*O*-glycans were derivatized using the in-solution SALSA procedure prior to cleavage from proteins. A protein fraction (500 µg) from salmon eggs was directly derivatized in a linkage-specific manner by adding premixed Reagents A and B from the SialoCapper™-ID Kit and incubating for 1 h at room temperature with shaking. Subsequently, SialoCapper Reagent C was added, and the reaction mixture was incubated for 3 min with shaking. To remove excess condensation reagents, SALSA-derivatized glycoproteins were precipitated by adding 5-fold ACN. Supernatants and precipitated proteins were separated by centrifugation (14,000× *g*, 20 min, 4 °C). The pellets were resuspended in a 5-fold excess of ACN and centrifuged under the same conditions. Precipitated glycoproteins (500 µg) were treated with 100 µL of 560 mM NaOH and 800 mM PMP in 50% MeOH and heated at 105 °C for 2 h in an open system [21]. After the BEP reaction, bis-PMP-labeled GN4 (200 pmol) was added as an external standard. The reaction mixture was neutralized with HCl and extracted with chloroform/water, and the aqueous layer was washed three times with chloroform. Subsequently, the aqueous layer was loaded onto a C18 solid-phase extraction column and washed with 0.1% formic acid and water. *O*-Glycan derivatives were eluted with 20 and 50% ACN and concentrated using a centrifugal evaporator.

#### 4.2.5. MALDI Matrix Preparation and Measurement Using MALDI-TOF/TOF MS

DHB (10 mg) was dissolved in 50% MeOH (1 mL) and mixed with 100 mM sodium chloride (NaCl) solution (30 µL). Samples were deposited onto a polished steel MALDI target by mixing the analyte and matrix solutions (1 µL each) on the target and enabling the mixture to dry by evaporation. All measurements were performed using a Rapiflex MALDI-TOF/TOF MS equipped with a Smartbeam 3D Nd:YAG laser pulsed at 355 nm (Bruker Daltonics, Bremen, Germany) and controlled using FlexControl 4.2 software, following general protocols. Spectra were obtained in reflectron mode under ion source voltage = 20 kV, pulsed ion extraction voltage = 2.57 kV, lens voltage = 11.65 kV, Reflector 1 voltage = 20.85 kV, Reflector 2 voltage = 1.085 kV, Reflector 3 voltage = 8.70 kV, and pulsed ion extraction delay = 160 ns. For MALDI-TOF/TOF MS measurements, the precursor ions were accelerated to 8 kV and selected using a timed ion gate. The fragments were further accelerated at 19 kV in the LIFT cell, and their masses were analyzed after ion reflector passage. No additional collision gas was used. Relative quantitative analysis was performed by comparing the areas of MS signals derived from each glycan and a known amount of the internal standard (A2GN1: 100 pmol and GN4: 40 pmol/100 µg of protein).

## Figures and Tables

**Table 1:** *N*-Glycan list in unfertilized *Oncorhynchus keta* eggs in positive ion mode.

No.	Putative Composition Candidate	GlyCosmos ID	Theor *m*/*z*	Obs *m*/*z*	pmol/100 µg of Proteins	±SD (*n* = 3)
N-1	(Man)2(GlcNAc)2	G03717EM	771.27	771.21	19.89	4.50
N-2	(Hex)2(HexNAc)2 + (SO_3_Na)1	G57583IJ	873.21	873.13	3.33	1.13
N-3	(Man)3(GlcNAc)2	G22768VO	933.32	933.25	74.92	16.31
N-4	(HexNAc)1 + (Man)2(GlcNAc)2	G10133VD	974.35	974.27	5.08	1.12
N-5	(Hex)3(HexNAc)2 + (SO_3_Na)1	G97679LA	1035.26	1035.19	6.05	1.10
N-6	(Hex)1 + (Man)3(GlcNAc)2	G38407UP	1095.37	1095.29	2.50	0.48
N-7	(HexNAc)1 + (Man)3(GlcNAc)2	G65343UJ	1136.40	1136.32	70.46	11.45
N-8	(Hex)4(HexNAc)2 + (SO_3_Na)1	G01561EA	1197.31	1197.23	1.47	0.23
N-9	(HexNAc)1 + (Man)3(GlcNAc)2 + (SO_3_Na)1	G65519YV	1238.34	1238.25	38.78	0.27
N-10	(Hex)2 + (Man)3(GlcNAc)2	G90206GU	1257.42	1257.34	3.86	0.11
N-11	(Hex)1(HexNAc)1 + (Man)3(GlcNAc)2	G17410PS	1298.45	1298.36	16.07	2.98
N-12	(HexNAc)2 + (Man)3(GlcNAc)2	G39943KJ	1339.48	1339.39	30.13	4.63
N-13	(Hex)1(HexNAc)1 + (Man)3(GlcNAc)2 + (SO_3_Na)1	G42901NB	1400.39	1400.30	9.16	1.66
N-14	(Hex)3 + (Man)3(GlcNAc)2	G45864TJ	1419.48	1419.39	2.51	0.21
N-15	(HexNAc)2 + (Man)3(GlcNAc)2 + (SO_3_Na)1	G86879HA	1441.42	1441.32	37.90	1.63
N-16	(Hex)2(HexNAc)1 + (Man)3(GlcNAc)2	G06928XT	1460.50	1460.41	2.00	0.80
N-17	(HexNAc)2(Fuc)1 + (Man)3(GlcNAc)2	G83355KE	1485.54	1485.44	1.75	0.14
N-18	(Hex)1(HexNAc)2 + (Man)3(GlcNAc)2	G02561FC	1501.53	1501.44	24.44	4.41
N-19	(HexNAc)3 + (Man)3(GlcNAc)2	G91972CK	1542.56	1542.46	21.71	0.66
N-20	(HexNAc)2 + (Man)3(GlcNAc)2 + (SO_3_Na)2	G52294SE	1543.36	1543.26	4.48	0.46
N-21	(Hex)4 + (Man)3(GlcNAc)2	G63923YN	1581.53	1581.43	1.82	0.26
N-22	(Hex)1(HexNAc)1(3,8NeuAc)1 + (Man)3(GlcNAc)2	G09057YJ	1602.58	1602.48	4.16	0.75
N-23	(Hex)1(HexNAc)2 + (Man)3(GlcNAc)2 + (SO_3_Na)1	G11415HH	1603.47	1603.37	22.24	3.97
N-24	(HexNAc)3 + (Man)3(GlcNAc)2 + (SO_3_Na)1	G83434SC	1644.50	1644.40	0.99	0.16
N-25	(Hex)1(HexNAc)2(Fuc)1 + (Man)3(GlcNAc)2	G50636SI	1647.59	1647.49	1.26	0.14
N-26	(Hex)2(HexNAc)2 + (Man)3(GlcNAc)2	G22140GZ	1663.58	1663.48	13.49	4.95
N-27	(HexNAc)3(Fuc)1 + (Man)3(GlcNAc)2	G75698JE	1688.62	1688.51	2.30	0.56
N-28	(Hex)1(HexNAc)3 + (Man)3(GlcNAc)2	G67992SW	1704.61	1704.51	10.07	1.53
N-29	(Hex)1(HexNAc)2 + (Man)3(GlcNAc)2 + (SO_3_Na)2	G93837PE	1705.41	1705.31	3.42	0.39
N-30	(Hex)5 + (Man)3(GlcNAc)2	G88433PA	1743.58	1743.48	1.59	0.16
N-31	(Hex)2(HexNAc)2 + (Man)3(GlcNAc)2 + (SO_3_Na)1	G30770WC	1765.52	1765.42	7.06	3.03
N-32	(Hex)1(HexNAc)2(3,8NeuAc)1 + (Man)3(GlcNAc)2	G28839WC	1805.66	1805.55	4.19	0.89
N-33	(Hex)2(HexNAc)2(Fuc)1 + (Man)3(GlcNAc)2	G36836GD	1809.64	1809.53	1.72	0.29
N-34	(Hex)1(HexNAc)3(Fuc)1 + (Man)3(GlcNAc)2	G58949BL	1850.67	1850.56	2.50	0.64
N-35	(Hex)2(HexNAc)3 + (Man)3(GlcNAc)2	G43761WH	1866.66	1866.55	10.59	4.44
N-36	(Hex)2(HexNAc)2 + (Man)3(GlcNAc)2 + (SO_3_Na)2	G85880TT	1867.46	1867.35	2.08	0.32
N-37	(Hex)6 + (Man)3(GlcNAc)2	G44306ED	1905.64	1905.55	1.18	0.19
N-38	(Hex)1(HexNAc)2(3,8NeuAc)1 + (Man)3(GlcNAc)2 + (SO_3_Na)1	G15739UL	1907.60	1907.51	2.96	0.72
N-39	(Hex)2(HexNAc)2(3,8NeuAc)1 + (Man)3(GlcNAc)2	G36598FV	1967.71	1967.60	5.29	1.37
N-40	(Hex)1(HexNAc)3(3,8NeuAc)1 + (Man)3(GlcNAc)2	G82602XV	2008.74	2008.62	1.55	0.32
N-41	(Hex)2(HexNAc)3(Fuc)1 + (Man)3(GlcNAc)2	G20218ZS	2012.72	2012.61	1.90	0.36
N-42	(Hex)3(HexNAc)3 + (Man)3(GlcNAc)2	G68164MW	2028.72	2028.60	1.07	0.26
N-43	(Hex)2(HexNAc)2(3,8NeuAc)1 + (Man)3(GlcNAc)2 + (SO_3_Na)1	G70896SI	2069.65	2069.53	2.62	0.78
N-44	(Hex)1(HexNAc)2(3,8NeuAc)2 + (Man)3(GlcNAc)2	G39207BN	2109.79	2109.66	1.45	0.41
N-45	(Hex)2(HexNAc)3(3,8NeuAc)1 + (Man)3(GlcNAc)2	G40734VV	2170.79	2170.66	4.53	3.84
N-46	(Hex)2(HexNAc)2(3,8NeuAc)2 + (Man)3(GlcNAc)2	G34449FW	2271.84	2271.70	3.84	1.62
N-47	(Hex)2(HexNAc)2(3,8NeuAc)2 + (Man)3(GlcNAc)2 + (SO_3_Na)1	G92758ZX	2373.78	2373.64	1.05	0.31
N-48	(Hex)2(HexNAc)2(Fuc)1(3,8NeuAc)2 + (Man)3(GlcNAc)2	G72956NR	2417.90	2417.75	0.63	0.16
N-49	(Hex)2(HexNAc)3(3,8NeuAc)2 + (Man)3(GlcNAc)2	G42989XH	2474.92	2474.77	1.46	1.15

The red text in the GlyCosmos ID indicates that the structure was identified/registered in this study.

**Table 2:** *O*-glycan list in unfertilized *Oncorhynchus keta* eggs in positive ion mode.

No.	Putative Composition Candidate	GlyCosmos ID	Theor *m*/*z*	Obs *m*/*z*	pmol/100 µg of Protein	±SD (*n* = 3)
O-1	(HexNAc)1	G57321FI	574.23	574.24	3.16	0.20
O-2	(Hex)1(HexNAc)1	G54499HR	736.28	736.28	20.92	2.32
O-3	(Hex)2(HexNAc)1	G70693FX	898.34	898.32	2.48	0.04
O-4	(Hex)1(HexNAc)2	G02368JK	939.36	939.35	0.98	0.07
O-5	(Hex)2(HexNAc)1(Fuc)1	G06967GN	1044.39	1044.38	3.80	0.37
O-6	(Hex)1(HexNAc)1(6NeuAc)1	G56682BC	1068.44	1068.43	3.25	0.11
O-7	(Hex)1(HexNAc)1(6NeuGc)1	G64851ON	1084.44	1084.42	19.89	1.06
O-8	(Hex)2(HexNAc)2	G69723CB	1101.41	1101.40	2.97	0.17
O-9	(Hex)2(HexNAc)2(Fuc)1	G58753MN	1247.47	1247.46	5.60	0.40
O-10	(Hex)3(HexNAc)2	G84791PR	1263.47	1263.45	5.71	0.28
O-11	(Hex)1(HexNAc)1(6NeuAc)1(3,8NeuAc)1	G97160DA	1372.57	1372.56	0.76	0.15
O-12	(Hex)1(HexNAc)1(6NeuAc)1(3,8NeuGc)1	G56882GN	1388.56	1388.55	3.29	0.31
O-13	(Hex)1(HexNAc)1(6NeuGc)1(3,8NeuGc)1	G34824ZD	1404.56	1404.54	18.53	0.50
O-14	(Hex)3(HexNAc)3	G16696H	1466.55	1466.53	1.96	0.29
O-15	(Hex)2(HexNAc)1(6NeuGc)1(3,8NeuGc)1	G38967LN	1566.61	1566.60	1.14	0.26
O-16	(Hex)2(HexNAc)2(Fuc)1(6NeuGc)1	G93628CF	1595.63	1595.61	1.35	0.27
O-17	(Hex)1(HexNAc)1(6NeuGc)1(3,8NeuGc)1(3,8NeuAc)1	G83831LP	1708.68	1708.67	0.51	0.17
O-18	(Hex)1(HexNAc)1(6NeuGc)1(3,8NeuGc)2	G21582IP	1724.68	1724.67	2.36	0.52
O-19	(Hex)2(HexNAc)2(6NeuGc)1(3,8NeuGc)1	G62787LM	1769.69	1769.68	0.57	0.21
O-20	(Hex)2(HexNAc)2(Fuc)1(6NeuGc)1(3,8NeuAc)1	G99337MF	1899.75	1899.74	0.12	0.10
O-21	(Hex)2(HexNAc)2(Fuc)1(6NeuGc)1(3,8NeuGc)1	G56538NZ	1915.75	1915.74	1.72	0.53
O-22	(Hex)1(HexNAc)1(6NeuGc)1(3,8NeuGc)3	G87232VO	2044.80	2044.79	0.53	0.22
O-23	(Hex)2(HexNAc)2(Fuc)1(6NeuGc)1(3,8NeuGc)2	G40207OL	2235.87	2235.85	0.58	0.26
O-24	(Hex)2(HexNAc)2(Fuc)1(6NeuGc)1(3,8NeuGc)3	G53493TB	2555.99	2555.97	0.31	0.16
O-25	(Hex)2(HexNAc)2(Fuc)1(6NeuGc)1(3,8NeuGc)4	G11613VV	2876.11	2876.09	0.13	0.10
O-26	(Hex)2(HexNAc)2(Fuc)1(6NeuGc)1(3,8NeuGc)5	G39946CZ	3196.24	3196.21	0.06	0.05
O-27	(Hex)2(HexNAc)2(Fuc)1(6NeuGc)1(3,8NeuGc)6	G33407XS	3516.36	3516.37	0.03	0.03

The red text in the GlyCosmos ID that the structure was identified/registered in this study.

## Data Availability

The data presented in this study are available upon request from the corresponding authors. The MALDI-TOF-MS data have been deposited in GlycoPOST (Accession No. GPST000706).

## Supplementary Materials

The following supporting information can be downloaded at: https://www.mdpi.com/article/10.3390/ijms27104646/s1.
