# Supplementary material for: N- and O-glycans in Unfertilized Chum Salmon (Oncorhynchus keta) Eggs Using Glycomic Techniques
Source: Int J Mol Sci. 2026 May 21;27(10):4646. doi: 10.3390/ijms27104646 (PMC13207093; doi:10.3390/ijms27104646)
Supplement: Supplementary file 1 [file ijms-27-04646-s001.zip › Supporting Information_Kurogochi 260521.pdf]

N- and *O*-glycans in Unfertilized Chum Salmon (*Oncorhynchus keta*) Eggs Using Glycomic Techniques

Masaki Kurogochi[1,*], Kai Suzuki[1,2], Di Wu[1,2], Hisatoshi Hanamatsu[1], Ken Kitajima[1],

Chihiro Sato[1,2] and Jun-ichi Furukawa[1,3,*]

Table S1. Glycan list of *N*-glycan in unfertilized *Oncorhynchus keta* eggs in negative mode.

No.	sulfate	putative composition candidate	GlyCosmos ID	Theor *m/z*	Obs *m/z*	pmol/100 μg of proteins	±SD (n=3)
N-2	1	(Man)2(GlcNAc)2+(SO$_3^-$)1	G57583IJ	827.23	827.09	3.49	1.46
N-5	1	(Man)3(GlcNAc)2+(SO$_3^-$)1	G97679LA	989.28	989.17	4.40	1.62
N-8	1	(Hex)4(HexNAc)2+(SO$_3^-$)1	G01561EA	1151.33	1151.24	0.93	0.21
N-9	1	(HexNAc)1+(Man)3(GlcNAc)2+(SO$_3^-$)1	G65519YV	1192.36	1192.27	38.78	2.24
N-13	1	(Hex)1(HexNAc)1+(Man)3(GlcNAc)2+(SO$_3^-$)1	G42901NB	1354.41	1354.32	7.10	0.46
N-15	1	(HexNAc)2+(Man)3(GlcNAc)2+(SO$_3^-$)1	G86879HA	1395.44	1395.35	38.65	1.92
N-20	2	(HexNAc)2+(Man)3(GlcNAc)2+(SO$_3$Na)1(SO3$^-$)1	G52294SE	1497.38	1497.28	4.04	2.32
N-23	1	(Hex)1(HexNAc)2+(Man)3(GlcNAc)2+(SO$_3^-$)1	G11415HH	1557.49	1557.40	21.30	2.09
N-24	1	(HexNAc)3+(Man)3(GlcNAc)2+(SO$_3^-$)1	G83434SC	1598.52	1598.42	0.59	0.10
N-29	2	(Hex)1(HexNAc)2+(Man)3(GlcNAc)2+(SO$_3$Na)1(SO3$^-$)1	G93837PE	1659.43	1659.33	3.00	1.43
N-31	1	(Hex)2(HexNAc)2+(Man)3(GlcNAc)2+(SO$_3^-$)1	G30770WC	1719.54	1719.45	6.69	2.78
N-36	2	(Hex)2(HexNAc)2+(Man)3(GlcNAc)2+(SO$_3$Na)1(SO3$^-$)1	G85880TT	1821.48	1821.38	1.57	0.79
N-38	1	(Hex)1(HexNAc)2(3,8Neu5Ac)1+(Man)3(GlcNAc)2+(SO$_3^-$)1	G15739UL	1861.62	1861.52	3.03	1.31
N-43	1	(Hex)2(HexNAc)2(3,8Neu5Ac)1+(Man)3(GlcNAc)2+(SO$_3^-$)1	G70896SI	2023.67	2023.56	3.06	1.56
N-47	1	(Hex)2(HexNAc)2(3,8Neu5Ac)2+(Man)3(GlcNAc)2+(SO$_3^-$)1	G92758ZX	2327.80	2327.67	1.42	1.04
N-50	1	(Hex)1(HexNAc)3+(Man)3(GlcNAc)2+(SO$_3^-$)1	G12606SR	1760.57	1760.47	0.58	0.05
N-51	1	(Hex)2(HexNAc)3+(Man)3(GlcNAc)2+(SO$_3^-$)1	G93362UO	1922.62	1922.53	0.39	0.04
N-52	2	(Hex)1(HexNAc)2(3,8Neu5Ac)1+(Man)3(GlcNAc)2+(SO$_3$Na)1(SO3$^-$)1	G84315HT	1963.56	1963.45	0.42	0.11
N-53	2	(Hex)2(HexNAc)2(3,8Neu5Ac)1+(Man)3(GlcNAc)2+(SO$_3$Na)1(SO3$^-$)1	G46347LC	2125.61	2125.50	0.34	0.20
N-54	1	(Hex)1(HexNAc)2(3,8Neu5Ac)2+(Man)3(GlcNAc)2+(SO$_3^-$)1	G55060ZW	2165.74	2165.63	0.89	0.43

The red text in the GlyCosmos ID indicates that the structure was identified/registered in this study.

Since the internal standard of the neutral glycan is not detected in negative mode, each glycan was calculated from the largest N-9 value (38.78) obtained in positive mode.

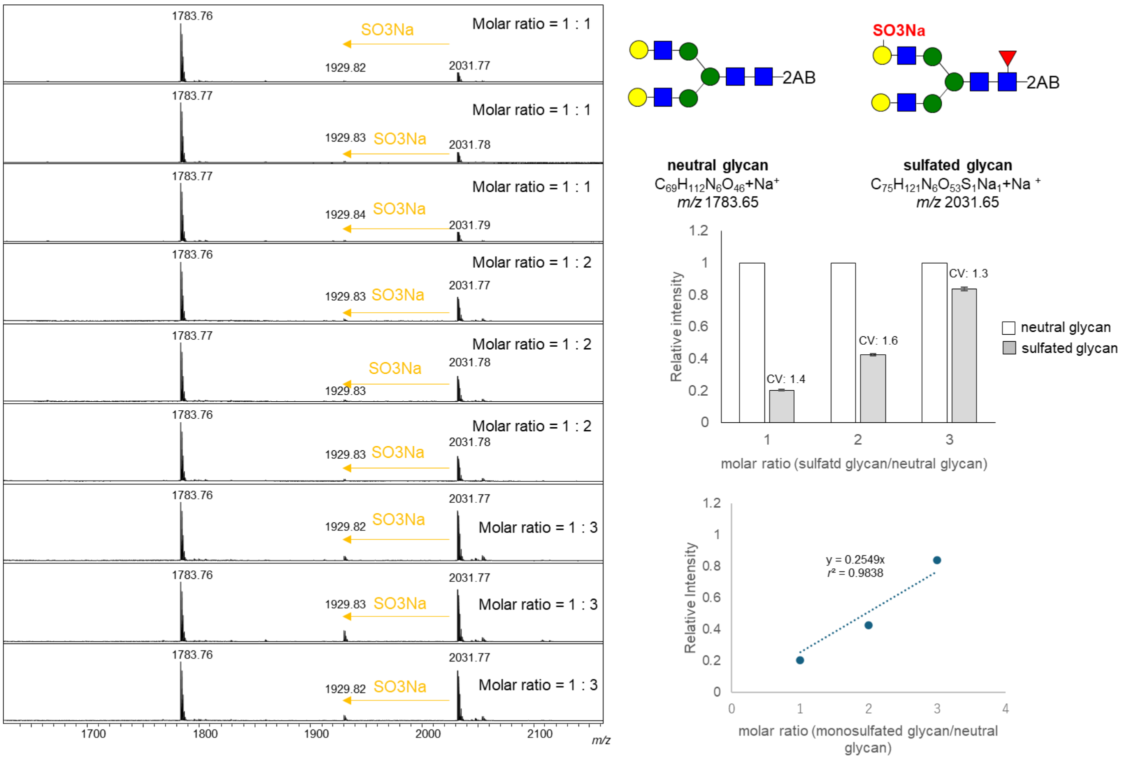

Figure S1. (A) MALDI-TOF MS spectra of neutral and monosulfated *N*-glycans mixed in different molar ratios (1:0, 1:2, 1:3) in triplicate. (B) Bar graph of relative intensities between neutral and monosulfated *N*-glycans. (C) Calibration curves (peak intensity ratio *vs*. molar ratio).

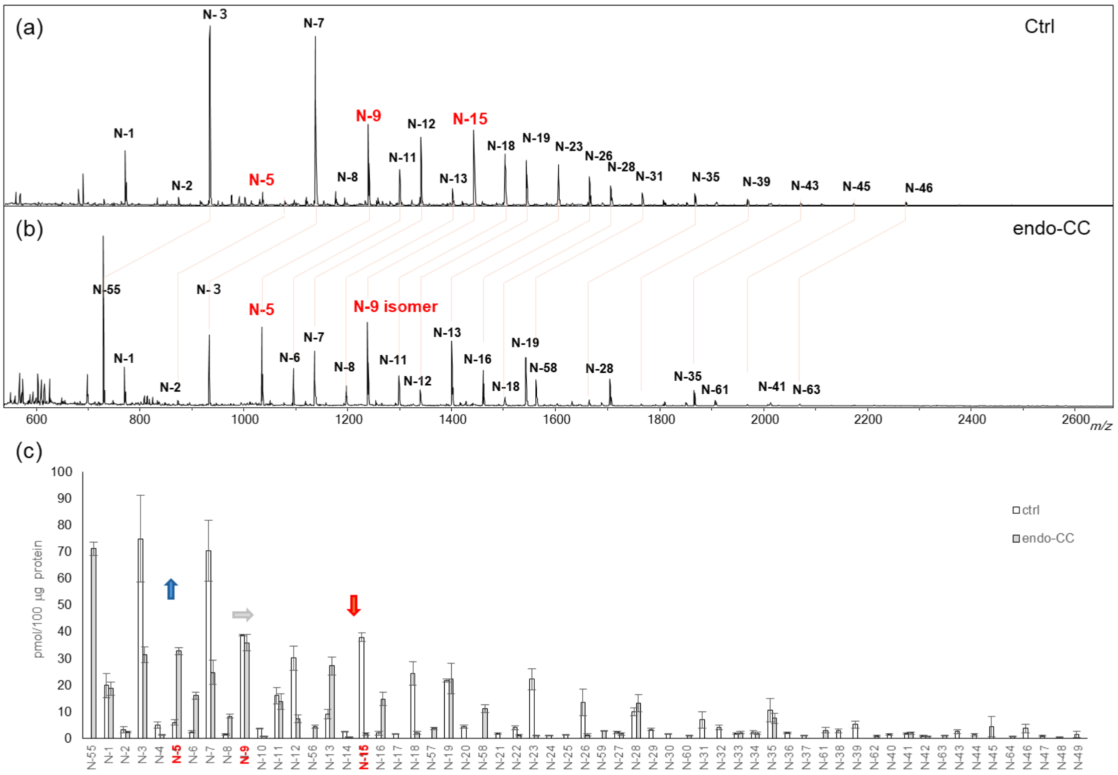

Figure S2. MALDI-TOF MS spectra showing *N*-glycans of the unfertilized *Oncorhynchus keta* eggs (a) without and (b) with endo-CC treatment. (c) Bar graph of signal intensities of N-glycans with and without endo-CC treatment. Signal numbers correspond to those described in Tables S1 and S3.

Table S2. Glycan list of *N*-glycan after endo-CC treatment in unfertilized *Oncorhynchus keta* eggs in positive ion mode (Figure S2a,b).

				Ctrl		endo-CC	
No.	putative composition candidate	Theor *m/z*	Obs *m/z*	pmol/100 μg of proteins	± SD (n=3)	pmol/100 μg of proteins	± SD (n=3)
N-55	(Hex)3(GlcNAc)1	730.24	730.21			71.13	2.37
N-1	(Hex)2(GlcNAc)2	771.27	771.23	19.89	4.50	18.69	2.42
N-3	(Hex)3(GlcNAc)2	933.32	933.28	74.92	16.31	31.34	2.91
N-5	(Hex)3(HexNAc)2+(SO_3Na)1	1035.26	1035.21	6.05	1.10	32.85	1.31
N-6	(Hex)4(HexNAc)2	1095.37	1095.33	2.50	0.48	16.09	1.41
N-7	(Hex)3(HexNAc)3	1136.4	1136.35	70.46	11.45	24.80	4.62
N-8	(Hex)4(HexNAc)2+(SO_3Na)1	1197.31	1197.26	1.47	0.23	8.39	0.82
N-9	(Hex)3(HexNAc)3+(SO3Na)1	1238.34	1238.29	38.79	0.27	35.97	3.13
N-11	(Hex)4(HexNAc)3	1298.45	1298.4	16.07	2.98	13.97	2.93
N-12	(Hex)3(HexNAc)4	1339.48	1339.43	30.13	4.63	7.37	1.57
N-56	(Hex)3(HexNAc)3+(SO3Na)2	1340.28	1340.23			4.44	0.65
N-13	(Hex)4(HexNAc)3+(SO3Na)1	1400.39	1400.34	9.16	1.66	27.29	3.40
N-15	(Hex)3(HexNAc)4+(SO3Na)1	1441.42	1441.36	37.90	1.63	1.72	0.41
N-16	(Hex)5(HexNAc)3	1460.5	1460.45	2.00	0.80	14.85	2.47
N-18	(Hex)4(HexNAc)4	1501.53	1501.48	24.44	4.41	2.26	0.58
N-57	(Hex)4(HexNAc)3+(SO3Na)2	1502.33	1502.28			3.79	0.50
N-19	(Hex)3(HexNAc)5	1542.56	1542.51	21.71	0.66	22.49	5.72
N-58	(Hex)5(HexNAc)3+(SO3Na)1	1562.44	1562.39			11.19	1.53
N-23	(Hex)4(HexNAc)4+(SO3Na)1	1603.47	1603.42	22.24	3.97	1.26	0.05
N-26	(Hex)5(HexNAc)4	1663.58	1663.53	13.49	4.95	1.34	0.36
N-59	(Hex)5(HexNAc)3+(SO3Na)2	1664.38	1664.33			2.88	0.21
N-27	(Hex)3(HexNAc)5(Fuc)1	1688.62	1688.56	2.30	0.56	1.74	0.53
N-28	(Hex)4(HexNAc)5	1704.61	1704.56	10.07	1.53	13.29	3.17
N-60	(Hex)5(HexNAc)4(3,8Neu5Ac)1	1764.63	1764.58			1.15	0.24
N-33	(Hex)5(HexNAc)4(Fuc)1	1809.64	1809.59	1.72	0.29	2.28	0.52
N-34	(Hex)4(HexNAc)5(Fuc)1	1850.67	1850.62	2.50	0.64	1.95	0.45
N-35	(Hex)5(HexNAc)5	1866.66	1866.61	10.59	4.44	7.59	1.83
N-61	(Hex)4(HexNAc)3(3,8Neu5Ac)2	1906.71	1906.65			3.16	1.04
N-62	(Hex)4(HexNAc)3(3,8Neu5Ac)2+(SO3Na)1	2008.64	2008.59			1.03	0.24
N-41	(Hex)5(HexNAc)5(Fuc)1	2012.72	2012.67	1.90	0.36	2.00	0.44
N-42	(Hex)6(HexNAc)5	2028.72	2028.66	1.07	0.26	0.82	0.24
N-63	(Hex)5(HexNAc)3(3,8Neu5Ac)2	2068.76	2068.7			1.07	0.22
N-64	(Hex)4(HexNAc)4(3,8Neu5Ac)2+(SO3Na)1	2211.72	2211.66			0.89	0.17

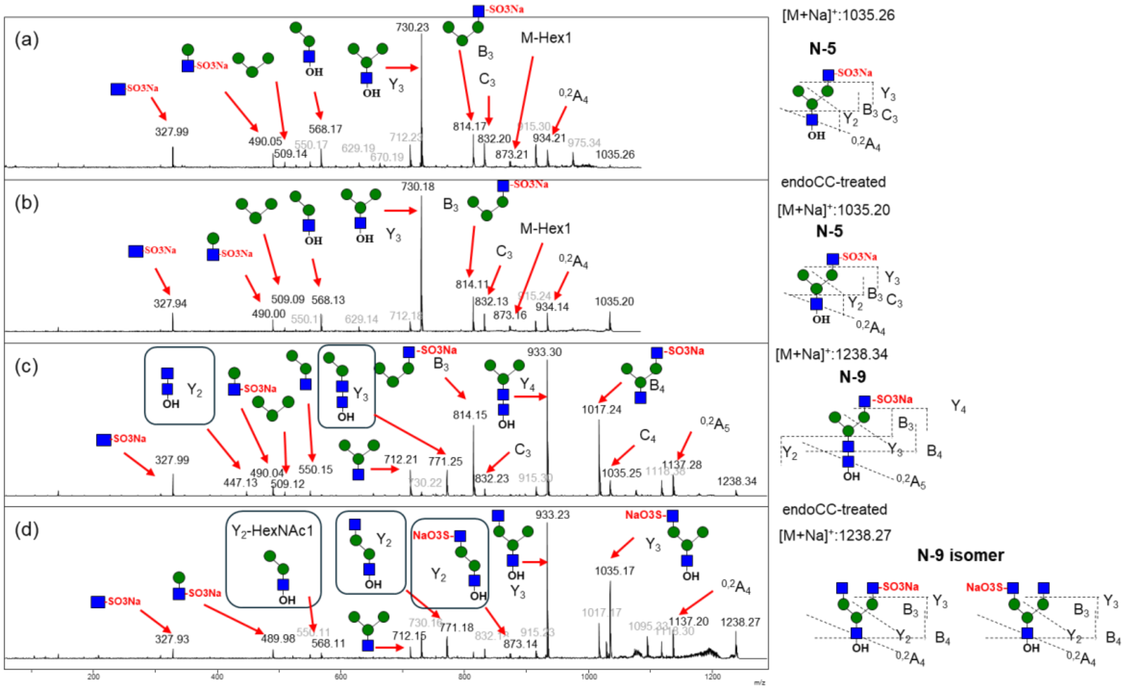

Figure S3. MALDI-TOF/TOF MS spectra of glycans at *m/z* 1035.26 (a), 1035.20 (b), 1238.34 (c), and 1238.27(d) in Figure S2.

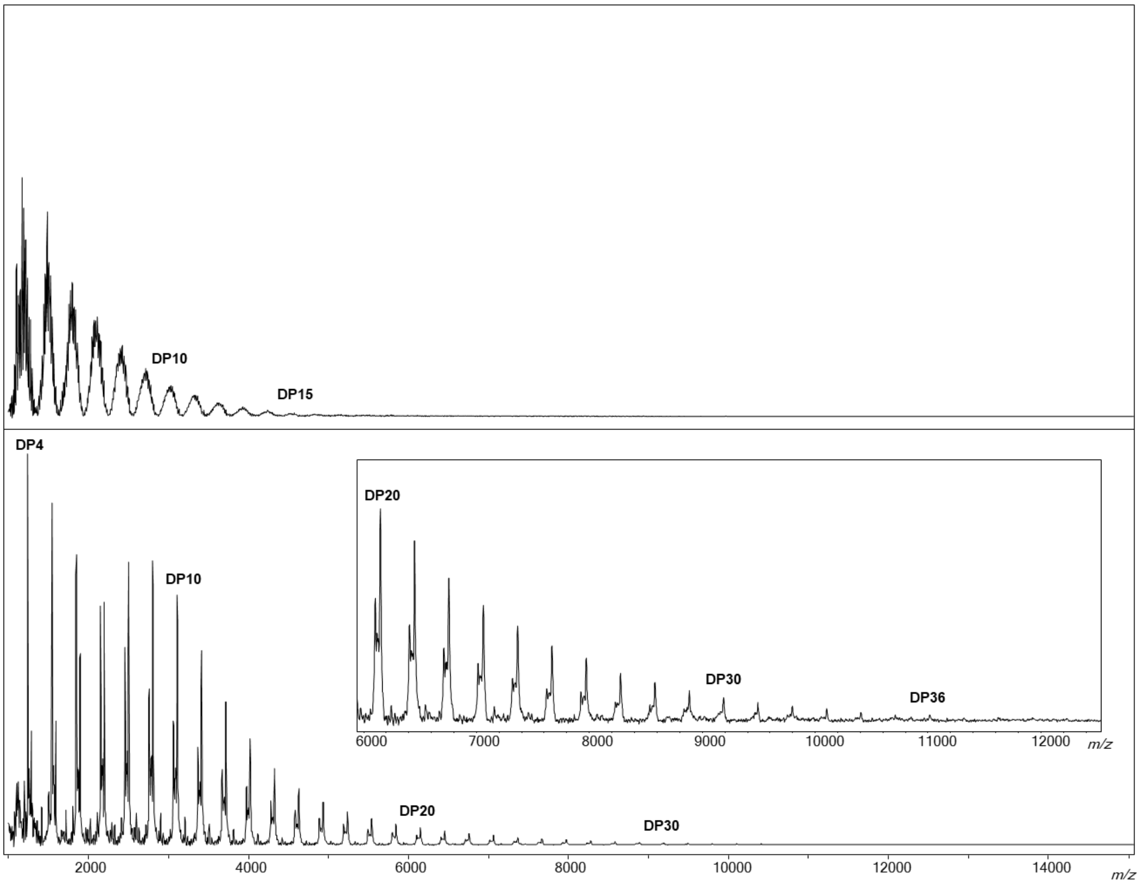

Figure S4. MALDI-TOF MS spectra showing (a) non-derivatized and (b) SALSA-derivatized colominic acid in linear mode.
